# Generation and Characterization of a Defective HIV-1 Virus as an Immunogen for a Therapeutic Vaccine

**DOI:** 10.1371/journal.pone.0048848

**Published:** 2012-11-07

**Authors:** Carmen Álvarez-Fernández, Alberto Crespo Guardo, Javier García-Pérez, Felipe García, Julia Blanco, Laura Escribà-García, Jose Maria Gatell, Jose Alcamí, Montserrat Plana, Sonsoles Sánchez-Palomino

**Affiliations:** 1 Institut dInvestigations Biomèdiques August Pi i Sunyer (IDIBAPS)-AIDS Research Group, Hospital Clinic, Catalonian Center for HIV Vaccines (HIVACAT) and University of Barcelona, Barcelona, Spain; 2 AIDS Immunopathology Unit. National Center of Microbiology, Instituto de Salud Carlos III, Madrid, Spain; 3 Institut de Recerca de la Sindrome de Inmunodeficencia Adquirida, IrsiCaixa, Badalona, Spain-HIVACAT; University of Pittsburgh, United States of America

## Abstract

**Background:**

The generation of new immunogens able to elicit strong specific immune responses remains a major challenge in the attempts to obtain a prophylactic or therapeutic vaccine against HIV/AIDS. We designed and constructed a defective recombinant virus based on the HIV-1 genome generating infective but non-replicative virions able to elicit broad and strong cellular immune responses in HIV-1 seropositive individuals.

**Results:**

Viral particles were generated through transient transfection in producer cells (293-T) of a full length HIV-1 DNA carrying a deletion of 892 base pairs (bp) in the *pol* gene encompassing the sequence that codes for the reverse transcriptase (NL4-3/ΔRT clone). The viral particles generated were able to enter target cells, but due to the absence of reverse transcriptase no replication was detected. The immunogenic capacity of these particles was assessed by ELISPOT to determine γ-interferon production in a cohort of 69 chronic asymptomatic HIV-1 seropositive individuals. Surprisingly, defective particles produced from NL4-3/ΔRT triggered stronger cellular responses than wild-type HIV-1 viruses inactivated with Aldrithiol-2 (AT-2) and in a larger proportion of individuals (55% *versus* 23% seropositive individuals tested). Electron microscopy showed that NL4-3/ΔRT virions display immature morphology. Interestingly, wild-type viruses treated with Amprenavir (APV) to induce defective core maturation also induced stronger responses than the same viral particles generated in the absence of protease inhibitors.

**Conclusions:**

We propose that immature HIV-1 virions generated from NL4-3/ΔRT viral clones may represent new prototypes of immunogens with a safer profile and stronger capacity to induce cellular immune responses than wild-type inactivated viral particles.

## Introduction

The generation of new immunogens able to elicit strong specific immune responses remains a major challenge in the attempts to obtain a prophylactic or therapeutic vaccine against HIV/AIDS [1], [2]. Whereas in the context of prophylactic vaccines the role of cellular responses in protecting against infection is unknown, in HIV-infected individuals the improvement of cellular HIV-specific immune responses appears to contribute to viral load control, both in animal models and clinical trials [3]–[7]. Strategies previously used to trigger strong cellular responses include immunization with defective viruses, inactivated viral particles, viral vectors expressing HIV-1 proteins and DNA [8], [9]. Inactivated viral particles display low immunogenicity due to modifications in protein structure and their lack of replicating capacity [10]. In contrast, live-attenuated virus vaccines have proved very successful in inducing strong cellular and humoral responses against SIV in macaque models [11]–[13]. However, for reasons of safety immunization with live-attenuated viruses is not recommended in the context of prophylactic vaccines due to the persistence of the attenuated virus in combination with ongoing low-level replication [14], [15]. In this setting, the error-prone replication machinery of the virus may eventually lead to the generation of wild-type pathogenic virus variants [16]–[18]. Alternatively, a virus that can execute only a single round of replication or conditionally live HIV-1 virus in which replication can be turned on and off at will can be used as a vaccine [19]–[22]. However, due to their limited replication, single-cycle virus vaccines of this kind may be less potent for the induction of protective immunity [15]. To improve safety, vaccine strains can be further attenuated through additional deletions or mutations in accessory genes or regulatory elements in order to reduce the pathogenicity of the virus [23]. Another approach could be to delete genes coding for critical enzymes involved in the viral cycle such as integrase or reverse transcriptase (RT). Recently, a DNA vaccine carrying a RT deletion has been assayed in macaques and although no protection was obtained, the induction of cellular immune responses led to decreased viral replication in vaccinated animals [24], [25]. The currently favoured subunit vaccines contain only a single or a few selected antigens due to the cost and manufacturing restrictions of producing a broad protein repertoire in their native structure. Virus-like particles (VLP) have recently emerged as an alternative to subunit vaccines, offering the advantage of mimicking the natural conformation of the capsid or envelope; they self-assemble into particulate structures, closely resembling the natural virus from which they are derived. VLPs are incapable of replication or infection, lacking regulatory proteins as well as infective genetic material [26].

Another approach to the development of therapeutic vaccines involves dendritic cell-based therapy, which is able to induce both primary and secondary immune responses of CD4+ and CD8+ T lymphocytes [27]–[29]. These cells present antigens not only in the MHC-class II pathway to helper CD4+ T cells but also in the MHC-class I pathway to cytotoxic CD8+ T lymphocytes [30], [31]. Several clinical trials based on dendritic cell immunotherapy for HIV infection have been reported [32]. Most of them found that DC immunotherapy elicits immunological responses, even though the design of the trials and the HIV antigens used to pulse dendritic cells were very different. Few of these trials have been performed with monocyte-derived dendritic cells from HIV-infected individuals which are cultured *in vitro* and pulsed with autologous viruses inactivated by different methods [33], [34]. Recently, in an uncontrolled study *Routy et al.* [35] reported the final results of an immunotherapy consisting of MD-DCs electroporated with mRNA encoding autologous HIV-1 antigens (Gag, Nef, Rev, Vpr). However, these studies have shown the induction of cellular responses and partial viral load control in some individuals [33]–[35].

The midterm success of these dendritic-cell therapeutic vaccines has been limited and, more importantly, they face major technical limitations. First, the generation of autologous viruses in Good Manufacturing Practices (GMP) conditions is expensive, time consuming and impractical when a large number of individuals is considered. Second, inactivation procedures such as heating result in protein degradation and decreased immunogenicity. Finally, inactivation methods decrease viral infectivity up to four logs, but residual viral activity remains [33], [34], [36]. To overcome these obstacles we have generated a defective recombinant virus based on the HIV-1 genome in which the RT is partially deleted (NL4-3/ΔRT). This vector generates infective but non-replicative particles when transfected in a producer cell line (293-T). We show that stimulating peripheral blood mononuclear cells (PBMCs) from HIV-infected individuals with NL4-3/ΔRT viral particles induces stronger cellular immune responses against HIV-1 than inactivated viral particles generated from a wild-type HIV-1 vector. The increased immunogenicity is probably related to the immature morphology of NL4-3/ΔRT virions. We propose that these non-replicative vectors could be used as safer immunogens for therapeutic immunization with dendritic cell-based HIV vaccines.

## Results

### Proviral NL4-3/ΔRT Clone Generates Infectious Non-replicative Viruses

**Figure 1:**
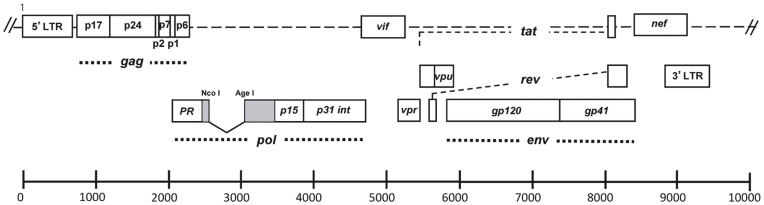
NL4-3/ΔRT plasmid graphic scheme. NL4-3 (WT) plasmid was used to generate the NL4-3/ΔRT plasmid by deleting an 892 bp fragment, which codes for the retrotranscriptase (RT) and is inside the *pol* gene. These plasmids were used to transfect 293-T cells in order to obtain viruses. Numbers refer to pNL4-3.

**Figure 2:**
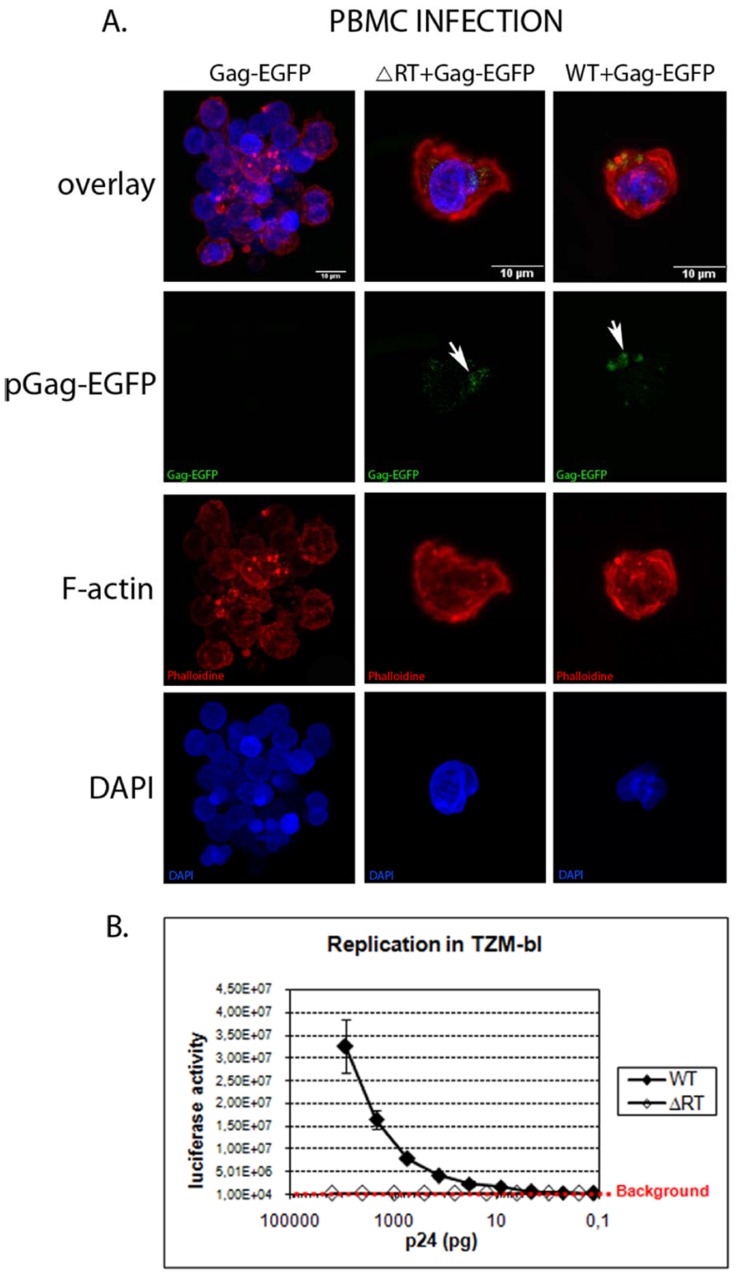
NL4-3/ΔRT plasmid renders virions infectious but non-replicative. (A) PBMC infection was monitored using NL4-3 (WT) and NL4-3/ΔRT (ΔRT) virions labelled with a Gag fusion protein (Gag-EGFP). Samples were imaged using a Leica SP2 confocal microscope. We observed an accumulation of Gag-EGFP in the cells infected with NL4-3 and NL4-3/ΔRT (arrows) but not in cells infected with Gag-EGFP. Cellular nuclei are stained in blue (DAPI), F-actin in red (Phalloidine) and the fusion protein Gag in green (Gag-EGFP). (B) NL4-3 and NL4-3/ΔRT virion replication measurements by luciferase activity in TZM-bl cells. The NL4-3 virions showed a decrease in luciferase activity directly proportional to the p24 quantity, while for NL4-3/ΔRT virions, luciferase activity was under the background independently of the p24 quantity.

Defective particles were produced by transient transfection of 293-T cells with a full length HIV-1 DNA carrying a deletion of 892 bp in the *pol* gene, encompassing the reverse transcriptase sequence (**Figure 1**). Viral production was examined at 48 or 72 hours post-transfection by an *in vitro* p24 ELISA assay and no significant difference was observed between pNL4-3 and pNL4-3/ΔRT transient transfection (data not shown). To evaluate the ability of NL4-3/ΔRT virions to promote the early steps of the retroviral replicative cycle, we produced virions carrying the Gag-EGFP fusion protein to monitor them. Confocal microscopy analysis of 293-T transiently transfected cells showed a cytoplasmic and membrane expression of Gag-EGFP (data not shown). Virions produced in cells expressing Gag-EGFP incorporated this fusion protein during their budding. PBMCs activated with IL-2 and PHA for two days were incubated with supernatant from 293-T transfected cells carrying virions labelled with Gag-EGFP. Three hours after incubation, PBMC were fixed, stained and analysed by confocal microscopy. In contrast to PBMCs incubated with pGag-EGFP supernatant, those incubated with NL4-3 plus Gag-EGFP or an NL4-3/ΔRT plus Gag-EGFP displayed EGFP punctuated areas (**Figure 2A**), showing that both NL4-3 and NL4-3/ΔRT virions were able to enter the cells.

The ability of the NL4-3/ΔRT to replicate was evaluated in TZM-bl cells, which carry a luciferase reporter under the control of the HIV-1 LTR promoter. Virus stocks prepared by transient transfection and normalized by the amount of p24 capsid protein were used to infect TZM-bl cells. Replication was determined by counting the luciferase activity of the cells three days post-infection. In contrast to NL4-3 virions, which show a replication correlative to the amount of p24 (**Figure 2B**), NL4-3/ΔRT virions were found to be non-replicative regardless of the amount of p24.

These results showed that NL4-3/ΔRT virions were able to enter cells but unable to produce new progeny due to the absence of retrotranscriptase activity.

### NL4-3/ΔRT Virion Morphology

Most HIV-1 virions appear as 110 nm diameter spherical particles with an electron-dense ring at the periphery surrounding a translucent core (immature morphology particles). Briefly, after the release of the virion, free capsid protein, cleaved from Gag polyprotein by the viral protease, is associated into a conical core structure at the centre of the particle, and the electron-dense ring at the periphery disappears (mature morphology particles). To check if the absence of the retrotranscriptase protein alters virion morphology or structure, electron microscopy analysis of 293-T cells transfected with pNL4-3 and pNL4-3/ΔRT was performed.

**Figure 3:**
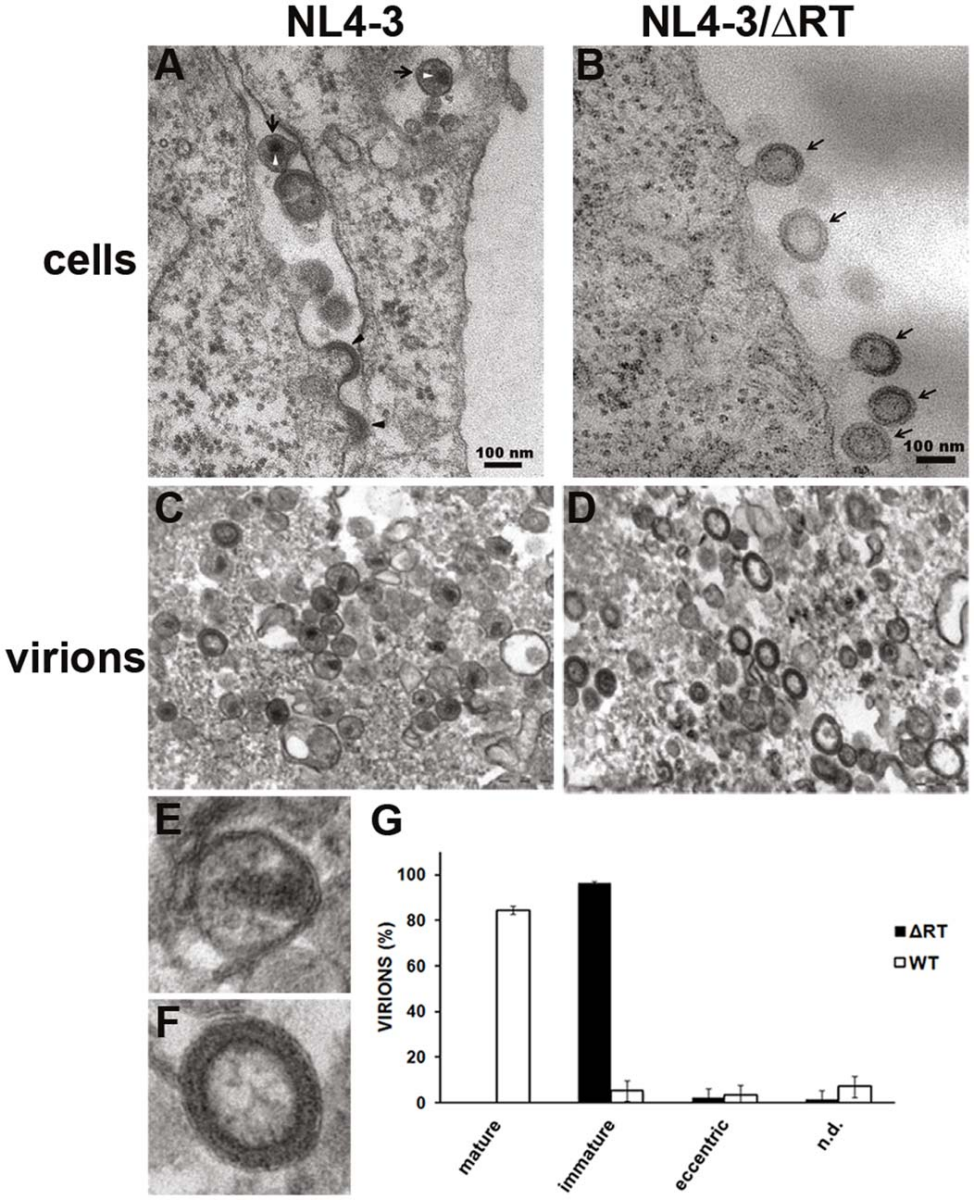
Analysis of virion morphologies **by electron microscopy.** (A) Electron micrographic analysis of 293-T cells transfected with pNL4-3. Mature virions (black arrows) show their characteristic core (white arrow heads). Budding particles (black arrow heads) are also present. (B) Electron micrograph showing immature viral particles budding from 293-T cells transfected with pNL4-3/ΔRT (black arrows). (C) Electron micrographic analysis of pNL4-3 purified viruses showing mature, immature and eccentric virion forms. (D) Electron micrograph of pNL4-3/ΔRT purified virions in which only immature virion forms can be observed. (E and F) NL4-3 and NL4-3/ΔRT virion magnifications respectively are shown. (G) Virion form measurements of NL4-3 *versus* NL4-3/ΔRT. NL4-3/ΔRT virion conformation was mostly immature and no mature virions were observed. On the other hand, NL4-3 virions were mainly mature. Error bars represent standard deviations. Samples were imaged using a Tecnai Spirit Twin electron-microscope.

Two days post-transfection with pNL4-3, electron micrographs showed the characteristic mature viral particles as well as budding virions (**Figure 3A**). However, the morphology of NL4-3/ΔRT virions close to the plasma membrane or during their budding was spherical, with two electron-dense layers surrounding a translucent centre, clearly resembling immature particles (**Figure 3B**). To confirm the immature morphology of NL4-3/ΔRT virions, we analysed ultracentrifugated purified virions by electron microscopy. Four hundred and sixty NL4-3 particles from 10 different micrographs were quantified and 85% showed the typical mature morphology with the canonical core structure in the middle (**Figure 3E**), the remaining 15% being immature and eccentric virions (**Figures 3C and 3G**). In contrast, 97% of the 585 NL4-3/ΔRT purified particles quantified in 10 different micrographs were immature viruses with a spherical shape surrounded by a ring of two electron-dense layers (**Figure 3F**). The rest of the particles could be classified as eccentric and non-determined forms, no mature particles were observed (**Figures 3D and 3G**).

**Figure 4:**
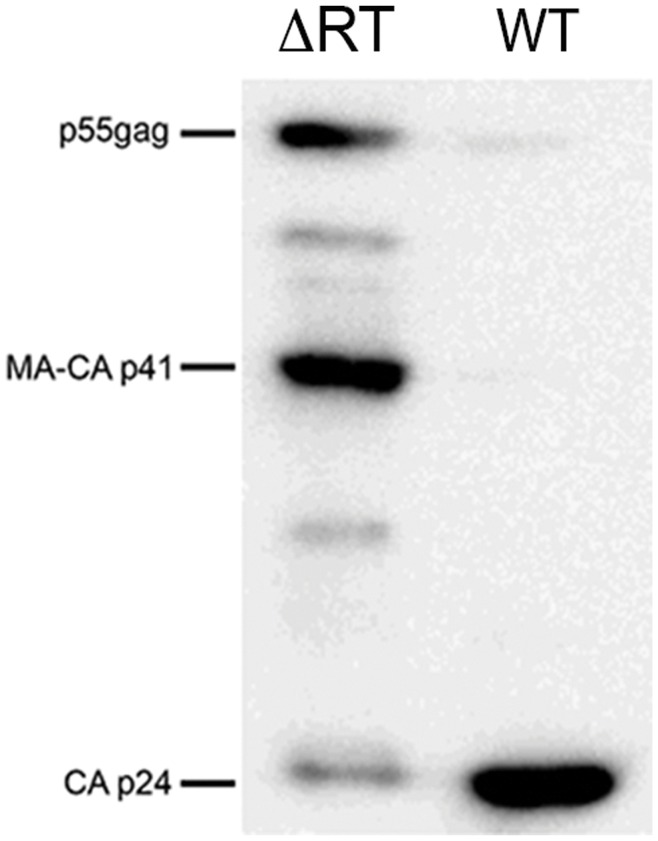
Protein profile of NL4-3 and NL4-3/ΔRT virions. Virions purified by ultracentrifugation were used to extract proteins and separated in a gradient 4-12% Tris-Glycine gel. An anti-p24 monoclonal antibody was used to study the processing profile of *Gag* in NL4-3/ΔRT and NL4-3 virions. There is a weaker processing of *Gag* in NL4-3/ΔRT virions, represented by an increase in p55-*gag* and MA-CA p41 forms in detriment of CA p24. At the same, an increase in intermediate forms is also shown in NL4-3/ΔRT virions.

The immature morphology of NL4-3/ΔRT virions suggested a problem in the Gag polyprotein processing by the viral protease. Western blot analysis of equal quantities of NL4-3 and NL4-3/ΔRT purified virions was performed to evaluate the viral processing. Using an anti-p24 monoclonal antibody, we confirmed that the processing of Gag polyprotein in NL4-3 purified virions was complete as shown by the presence of p24 and the loss of nearly complete p55-*gag* and MA-CA-p41 (**Figure 4**). NL4-3/ΔRT purified virions processed Gag polyprotein into CA p24; however, intermediate forms such as p55-*gag* and MA-CA-p41 were still present, suggesting an incomplete processing that could explain the immature morphology of the virions.

### NL4-3/ΔRT Virions were able to Induce Specific-cellular Immune-responses

**Figure 5:**
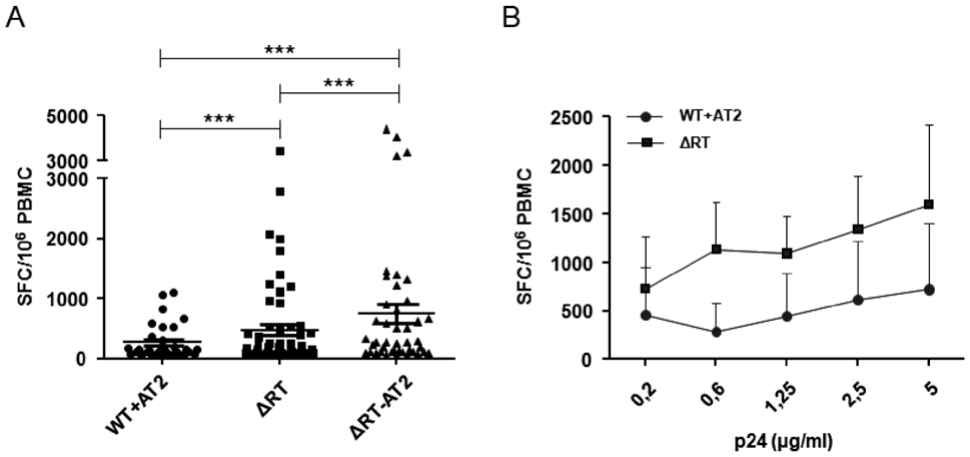
Immunogenicity of different viral constructions measured by ELISPOT responses. (A) Cryopreserved PBMCs from asymptomatic HIV seropositive individuals were tested for specific IFN-γ secreting T cells by *in vitro* stimulation with different HIV virions (WT+AT-2, ΔRT and ΔRT+AT-2). PBMCs were pulsed with 200 ng/ml p24 equivalents in all cases. The response elicited by WT+AT-2, ΔRT and ΔRT+AT-2 virions (mean ± SEM) is shown. The positivity threshold for each construct or antigen was defined as at least 50 SFC/10^6^ PBMC and at least twice that of the control medium. The magnitude of response differed significantly between stimuli (***p<0.001). (B) Four responder individuals were tested with different concentrations of WT+AT-2 and ΔRT virions (from 200 ng/ml to 5 µg/ml). The titration showed that the difference found between both stimuli was maintained, independently of the amount of virions used.

We assessed the immunogenic capacity of NL4-3/ΔRT virions by a classical ELISPOT assay evaluating the presence of T-specific IFN-γ producing cells by testing a panel of PBMC samples from chronic asymptomatic HIV-infected individuals (n = 69). In parallel, we also used Aldrithiol-2 (AT-2) inactivated NL4-3 and NL4-3/ΔRT virions, which did not produce replicative viruses. To rule out false-positive T-cell responses and to confirm the specificity of our approach we also performed ELISPOT assays using samples from healthy HIV-uninfected individuals and preparations of viral particles obtained from supernatants of 293-T cells transfected with irrelevant plasmids. As expected, no positive response was detected in either case. Surprisingly, NL4-3/ΔRT defective particles triggered cellular responses in a larger proportion of individuals (55% of individuals) than AT-2 inactivated wild type NL4-3 viruses, in which case only 23% of individuals responded. It should be noted that the latter 23% responded to both AT-2 inactivated NL4-3 and NL4-3/ΔRT whereas an additional 32% of individuals only responded to NL4-3/ΔRT and not to AT-2 inactivated NL4-3 viruses. Moreover, AT-2 treatment did not affect the induction of cellular immune responses, since the number of individuals who showed positive responses (63%) was even higher. Evaluating the magnitude of the elicited immune response, we observed that the mean of the SFC/10^6^ PBMC obtained after exposure to NL4-3/ΔRT viruses was significantly higher than after using NL4-3 treated with AT-2 (454 vs 259 SFC/10^6^ PBMC respectively; p<0.005), even increasing the concentrations of both viruses (figures 5A and 5B). We also observed that the mean of the immune response elicited with AT-2 treated NL4-3/ΔRT virions was significantly higher (712 SFC/10^6^ PBMC) than with AT-2 inactivated NL4-3 viruses, and also higher than with NL4-3/ΔRT virions. This latter result may suggest that AT-2 treatment did not impair the immunogenicity of defective viruses and probably indicates that this chemical inactivation was not deleterious for the integrity of this viral construct.

### Effect of Amprenavir (APV) Virus Treatment on the Immunogenic Capacity

**Figure 6:**
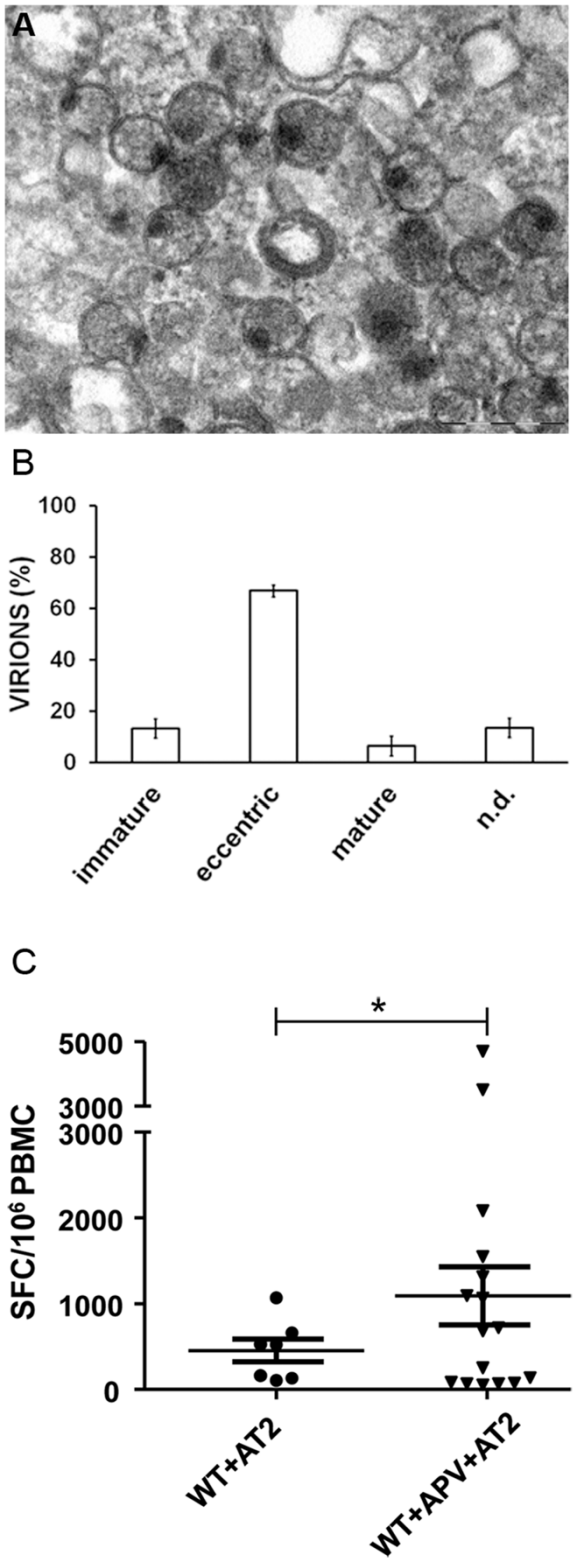
Immature NL4-3 virions improved the elicited cellular immune response. (A) Electron microscopy analysis of purified NL4-3 virions treated with AT-2 and APV. Different virion morphologies were observed; most were immature and eccentric. (B) 52 electron-micrographs were analysed for virus morphologies. The APV incubation reduces mature virions but increases immature and eccentric forms. Error bars represent standard deviaations. (C) Twenty-one cryopreserved PBMCs from HIV seropositive individuals were assessed by ELISPOT to detect IFN-γ production. PBMCs were pulsed with 200 ng/ml p24 equivalents in all cases. Magnitude response was significantly lower against WT inactivated virions (WT+AT-2) than against WT+APV+AT-2 (*p<0.05). Averaged values from duplicate wells normalized to SFC/10^6^ PBMCs are shown for the stimulation conditions indicated. Plots represent mean ± standard error of the mean (SEM). Positivity threshold for each construct or antigen was defined as at least 50 SFC/10^6^ PBMC and at least twice that of the control medium.

To assess whether the immaturity status of the defective viruses might be involved in their immunogenic capacity, we decided to generate immature NL4-3 viruses through treatment with the protease inhibitor APV, which has previously been shown to block virus infectivity and render particles immature [37]. Electron microscopy analyses showed that most of the particles had an eccentric immature morphology with an electron-dense patch on one side (**Figure 6A**). Six hundred and eighteen particles from 52 different micrographs showed that 67% of the particles appeared to be eccentric and 12% immature, compared with 7% that were mature (**Figure 6B**).

ELISPOT analysis (n = 21) was applied to AT-2 inactivated NL4-3 viruses generated either in the presence or absence of APV. The ability to generate an immune response was increased when using immature AT-2 inactivated NL4-3 viruses treated with APV (obtaining immune response in 67% *versus* 33%), being similar to the proportion of HIV-infected patients able to respond to NL4-3/ΔRT. In addition, analysing the response magnitude, we observed that the SFC/10^6^ PBMC mean was significantly lower (p<0.05) in the case of AT-2 inactivated NL4-3 viruses in comparison with AT-2 inactivated NL4-3 treated with APV (455 vs.1092 SFC/10^6^ PBMC) respectively (**Figure 6C**).

## Discussion

Current antiretroviral treatment can control viral load and immune recovery but combined Active Antiretroviral Therapy (cART) does not eradicate the virus [38]. Consequently, treatment must be maintained for the entire lifetime, with the risk of secondary effects and emergence of resistances, and at a high economic cost. Therapeutic vaccination, aiming at the induction of cellular immune responses able to control HIV replication, is an alternative to chronic cART [1]. Inactivated viruses, poxvirus vectors, virus-like particles and dendritic cell-based vaccines have all been assayed in clinical trials, albeit with limited success [9], [33], [36], [39].

Since reverse transcription of retroviral genomes into double stranded DNA is a key event for viral replication, we constructed and characterized a defective recombinant vector based on the HIV-1 genome in which only 892 bp of the RT gene were deleted, including RT fingers, palms and thumb subdomains (pNL4-3/ΔRT; **Figure 1**), to serve as immunogen candidate in therapeutic HIV vaccines. We demonstrate that RT deleted viral particles are able to infect target cells (**Figure 2A**) but do not allow a new cycle of replication in the infected cell (**Figure 2B**). The safety of viral constructs generating full viral genomes is a matter of major concern, particularly in the context of already infected individuals in whom recombination events may potentially occur. In the case of co-infection of the same cell by NL4-3/ΔRT particles and wild type-virus, trans-activity of reverse transcriptase from the latter could result in retrotranscription of the defective NL4-3/ΔRT RNA and generation of recombinant HIV-DNA susceptible to integration and subsequent replication. To avoid this possibility we performed the 892 bp deletion mentioned above that encompasses the thumb subdomain in the RT, which is required for the tRNALys3 packaging into HIV-1 [40]. The absence of the RT sequence avoids the formation of the tRNA/LysRS and blocks the annealing to the viral RNA [41]. This deletion therefore blocks the formation of the reverse transcription initiation complex, even when RT molecules from co-infecting wild type virus cells are present [42], [43]. As a complementary strategy to prevent recombination events, the administration of antiviral drugs at the same time as vaccination could be envisaged [44].

The use of other viral constructs like Integrase-defective lentiviruses has been proposed as a strategy for the safe immunization. *In vitro* and *in vivo* data [45], [46] show that these vectors display appropriate immunogenicity that is comparable to vaccination with wild type vectors. However, even though integration is blocked, reverse transcription and generation of HIV-DNA molecules are produced. It has recently been reported that exposure to cytoplasmic DNA products elicits antiviral responses leading to activation of caspases and massive apoptosis through a bystander phenomenon in non-infected lymphocytes [47].

Conversely, inhibition of early reverse transcription results in decreased toxicity through this pro-apoptotic mechanism. Therefore, compared to integrase-deleted viruses, our NL4-3ΔRT vectors, which do not allow retrotranscription and DNA synthesis, would improve the safety of the immunogen. We show that viral particles generated from NL4-3/ΔRT vectors are able to induce a significant immune response when used *in vitro* in PBMC cultures from infected individuals. Interestingly, when PBMCs from 69 chronic asymptomatic HIV-infected individuals were stimulated with NL4-3 and NL4-3/ΔRT viral particles, a higher number of subjects responded to NL4-3/ΔRT particles than to AT-2 inactivated NL4-3 virions (55% *versus* 23% individuals). Moreover the number of IFN-γ producing cells was increased in PBMCs stimulated with NL4-3/ΔRT virions. Therefore, NL4-3/ΔRT particles increased both the proportion of responders to HIV antigens and the magnitude of cellular responses. One possible to explanation for this finding is that AT-2 treatment may decrease the immunogenicity of viral particles [48]. However, when we tested this hypothesis through AT-2 inactivation of NL4-3/ΔRT particles, even a higher immunogenic activity was found, a result that rules out this mechanism. Another difference between NL4-3 and NL4-3/ΔRT virions that could explain the differences in immunogenicity was the immature morphology in the capsid. In fact, in electron microscopy analysis we observed that more than 97% of NL4-3/ΔRT viral particles displayed an immature morphology (**Figures 3B, D and F**). Differences in viral maturation was confirmed by Western blot of virions produced by wild type or NL4-3/ΔRT vectors, as shown by a sharp decrease in p55-*gag* processing and lower generation of mature CA p24 protein (**figure 4**). So far, the generation of immature particles has been documented after treatment with protease inhibitors, mutations of protease cleavage sites in Gag or by inhibitors of Gag processing [49]-[51]. However, it has been reported that mutations in the RT gene affect virion maturation and RNA dimer stability [52]. Besides, cleavage of p55-*gag* is required for RNA dimerization and further virion maturation [53]. Overall, these results suggest that viral RT sequences play a role in the control of protease activity and viral maturation, as we found in our experiments (**figure 4**). This mechanism could explain the generation of immature particles by our NL4-3/ΔRT constructs, which in turn, would further improve safety.

Interestingly, viral preparations produced by NL4-3/ΔRT vectors induced higher proportions of IFN-γ producing cells, raising the possibility that immature particles may be more immunogenic than wild-type virions. To test this hypothesis we generated virions from a wild-type HIV-1 genome in the presence of a protease inhibitor (APV) [37]. Immature virions with eccentric core morphology were observed by electron microscopy (**Figure 6A**) and these AT-2 inactivated viral preparations displayed stronger immunogenicity than AT-2 treated wild type viruses (NL4-3) generated in the absence of a protease inhibitor (**Figure 6C**). We therefore conclude that immature viral particles display higher potency as viral immunogens [54], [55] and that this is probably the underlying mechanism responsible for the stronger induction of cellular responses observed with NL4-3/ΔRT viruses. A possible explanation for this observation is that immature particles are more stable, increasing the exposure of important epitopes on their surface [56], leading to higher loads of viral proteins in presenting cells and increased abundance of peptides generated by proteolytic processing. Actually, previous reports have shown that dendritic cells can cross-present virus-like particles using an endosome-to-cytosol pathway [57], [58]. NL4-3/ΔRT immature particles may be preferentially directed towards this pathway of protein processing and antigen cross-presentation by specialized presenting cells that induce efficient immune responses in PBMC cultures [59], [60]. Overall, these data suggest that immature, defective non-replicative viruses may be good candidates as imunogens due to their greater capacity to induce specific immune responses.

Since HIV is a highly variable retrovirus, and since differences in sequence between two viruses within a subtype can be as high as 30%, the construction of consensus sequences has been proposed as an alternative to cover the required diversity of subtypes [61], [62] **.** The NL4-3/ΔRT recombinant vectors described here might carry optimized sequences of different HIV-1 genes in order to improve their immunogenicity.

In fact our group has developed a dendritic cell-based vaccine using autologous inactivated viruses that shows induction of cellular responses and moderate control of viral load in vaccinated subjects [34], [36]. The generation of autologous inactivated viruses at levels required for stimulating dendritic cells is a limiting and time-consuming step. To overcome these hurdles, after proper modification, the NL4-3/ΔRT based vectors described here could be used to generate viral chimeras through the insertion of genomic fragments amplified directly from plasma RNA or proviral DNA obtained from HIV infected individuals. This strategy would allow the production of immature, non-replicative chimeric viral particles to pulse dendritic cell cultures in order to induce a strong cellular response against autologous HIV epitopes.

However, our study has a number of drawbacks. First, despite the detection of significant levels of IFN-γ production in the presence of defective HIV-1 virions *in vitro*, we did not test the potential immunological impact of this viral preparation through other approaches (e.g. the study of T cell polyfunctionality). Second, the results were generated *in vitro* in a limited group of patients and they must be replicated in larger samples. Finally, the final goal of any therapeutic vaccine is to induce a potent immune response able to control viral replication in the absence of cART (“functional cure”) in a significant proportion of patients, and this objective needs to be further characterized in clinical trials.

Nonetheless, the present study showed that immature non-replicative HIV-1 virions generated from NL4-3/ΔRT represent effective and safe novel immunogens able to induce strong cellular responses against HIV in PBMC isolated from many HIV-1 infected individuals, and may therefore represent a promising strategy for improving the immunogenicity of therapeutic HIV vaccines.

## Materials and Methods

### Viral Vectors

NL4-3 plasmid was obtained from the NIH AIDS Research and Reference Reagent Program (NIH ARRRP, catalogue no. 114) [63]. pNL4-3/ΔRT was generated using the previously described pNL4-3LacZ/RTRen [64]. Briefly, this vector contains the *LacZ* gene cloned replacing the RT sequence (2,590 and 3,486 positions of pNL4-3) and the *renilla* reporter gene substituting *nef* (8,797 and 8,887 positions of pNL4-3). To generate the pNL4-3/ΔRT we reconstituted the *nef* gene by cloning 90 bp in NotI/XhoI restriction sites from pNL4-3. *LacZ* gene was deleted through NcoI/AgeI digestion and blunt-end ligation using the Klenow polymerase. The resulting 13,983 bp plasmid containing HIV-1 plus pUC19 nucleic sequences is termed pNL4-3/ΔRT (**Figure 1**) (patent code EP 11382103.7). The pGag-EGFP vector was obtained from Dr. G. Pavlakis through the NIH ARRRP (catalogue no.11468).

### Cell Culture

293-T cells were purchased from the American Type Culture Collection (ATCC, Rockville, MD) (CRL-11268). The TZM-bl cell line (expressing CD4 receptor and CCR5/CXCR4 co-receptors, with an integrated LTR-Luc reporter system) [65]–[68] was obtained from the NIH ARRRP (catalogue no. 8129). Both cell lines were maintained in Dulbecco’s modified Eagle’s medium (DMEM) supplemented with 10% heat inactivated fetal bovine serum (FBS), 2 mM glutamine, penicillin (100 U/ml) and streptomycin (100 µg/ml) (DMEM-10). All culture media reagents were from Invitrogen (Madrid, Spain).

PBMCs were isolated from buffy coats of healthy blood donors (Banc de Sang i Teixits, BST, Barcelona, Spain) by centrifugation through a ficoll-hypaque gradient. Primary cells were cultured in RPMI-1640 medium supplemented as described above (RPMI-10). These cell cultures were supplemented with 100 U/ml human interleukin (hIL)-2 (SIGMA-ALDRICH, Madrid, Spain) and 5 µg/ml phytohemagglutinin (PHA; SIGMA-ALDRICH) once every two weeks.

All cell cultures were performed at 37°C in a fully humidified atmosphere with 5% CO_2_ in air.

### Study Individuals

Samples of EDTA-anticoagulated venous blood samples were isolated from healthy blood donors and from treated and untreated chronic asymptomatic HIV-1 individuals with baseline CD4+ T lymphocyte counts >500 cells/mm^3^ and plasma viral loads ranging from 50–10,000 HIV-1 RNA copies/mL (Hospital Clinic, Barcelona, Spain), by centrifugation through a ficoll-hypaque gradient and cryopreserved.

All the individuals gave informed written consent and the study was reviewed and approved by the Institutional Ethical Committee Board of the Hospital Clinic (Barcelona, Spain).

### Generation of Virus Stocks

Viruses were produced by transient transfection in 293-T cells. Briefly, the previous day 1.5×10^6^ 293-T cells were seeded in 75 cm^2^ tissue culture flasks in DMEM-10 medium. Cells were replaced with fresh DMEM-10 medium three hours before transfection by the calcium-phosphate method (ProFection® Mammalian Transfection System; Promega, Madison, WI) according to the manufacturer’s instructions, using 5 µg of each DNA construct previously purified (Qiagen, Valencia, CA). Growth medium was replaced with fresh DMEM-10 medium 16–18 h post-transfection. For immature wild-type (wt) virion production, 1 nM APV, corresponding to the IC50 [69], was added to the medium. The supernatants were harvested approximately two days after transfecton and clarification by centrifugation at 3500 rpm/4°C for 10 minutes. Virus stocks were inactivated with AT-2 treatment when required. To this end, a 100 mM stock solution in DMSO (SIGMA-ALDRICH) was prepared and added directly to the virus to produce a 1 mM AT-2 solution and treated at 4°C overnight [48], [70].

Viral supernatants were concentrated through centrifugation at 28,000 rpm/4°C for 32 minutes (Sorvall Ultra Series WX Ultra 80; Thermo Fisher Scientific, Asheville, NC). The pellets were centrifuged once more at 40,000 rpm/4°C for 10 minutes and resuspended in 500 µl 1X PBS before being stored at −80°C. Viruses were quantified by determining the concentration of p24 in the supernatant with an antigen (Ag) capture assay (ELISA; Innogenetics NV, Gent, Belgium).

### *In vitro* Infectivity Assay

Viral infectivity (replication) was measured using TZM-bl marker cells that carry a *luciferase* gene under the control of the HIV-LTR. The expression of the *luciferase* gene in these cells is repressed by the absence of the Tat protein, and is activated by HIV-1 replication. Briefly, a 96-well plate was set up with 10^4^ cells/well in the viral stocks previously quantified by p24 antigen for testing. Viruses were loaded in triplicate with three sets of nine serial fourfold dilutions of NL4-3 and NL4-3/ΔRT viruses as well as three uninfected controls. The plate was then placed in a humidified chamber and cultured as described above. The supernatants were removed after three days of cell culture and the cell-associated luciferase activity for each well was determined on a microplate luminometer (Turner Biosystems, Sunnyvale, CA) by using a luciferase assay kit (BioTherma AB, Dalarb, Sweden).

### Confocal Microscopy

293-T cells were transiently transfected as described above with pGag-EGFP (5 µg) alone, pNL4-3 (3 µg) plus pGag-EGFP (2 µg) and pNL4-3/ΔRT (3 µg) plus pGag-EGFP (2 µg). Two days later, supernatants were collected and used for subsequent infection. Viruses were quantified by Ag p24. Two µg of p24 of NL4-3/Gag-EGFP and 2 µg of NL4-3/ΔRT/Gag-EGFP as well as 1 ml of Gag-EGFP transfection supernatant were added to 25×10^6^ PBMC. After 3–4 hours at 37°C, cells were centrifuged at 1,500 rpm for 5 minutes. Supernantants were discarded and cells fixed with paraformaldehyde 4% solution (SIGMA-ALDRICH), stained with DAPI (1/10,000; SIGMA-ALDRICH) and phalloidine (1/1,000; SIGMA-ALDRICH) for 30 minutes at room temperature, after being mounted in Vectashield mounting solution (Burlingame, CA). Images were captured using a Leica SP2 confocal microscope (Leica Microsystems, Wetzlar, Germany) and processed using ImageJ [http://rsbweb.nih.gov/ij/index.html].

### Electron Microscopy

Virus stocks were produced and concentrated as described above. Cells were cleaned with 1X PBS, the washing solutions were removed and the cell monolayers or pelleted viruses were fixed and kept in 10 ml of glutaraldehyde 2.5% in 1 M PB pH 7.4 at 4°C. After 20 minutes, cells were scraped and transferred to a 15 ml flask, and pelleted at 1,500 rpm for five minutes. Pellets were fixed and included in resin according to standard procedures [71]–[73]. Electron microscopy images were obtained using a Tecnai Spirit Twin (FEI, Hillsboro, OR) and processed using ImageJ [http://rsbweb.nih.gov/ij/index.html]. Morphology quantification of 460 NL4-3 and 585 NL4-3/ΔRT viruses was performed with 10 different micrographs of 12 µm^2^ each. Morphology quantification of 618 NL4-3+ APV particles was performed with 52 different micrographs of 0.6 µm^2^ each.

### Viral Protein Analysis

Purified virions were resuspended in lysis solution. After 15 minutes of incubation at 4°C, samples were centrifuged for 15 minutes at 13,000 rpm at 4°C. Supernatants were collected and kept at −20°C. Protein concentration was determined by Bradford reaction (Bio-Rad Laboratories, Madrid, Spain) [74].

For Western blot (WB) 200 ng of the different extracts quantified by Ag p24 were mixed with Laemmli buffer and resolved in pre-cast 4–12% Tris-Glycine gels (Novex® 4–12% Tris-Glycine Mini Gel; Invitrogen). PVDF membranes (Invitrogen) were activated for 10 minutes in methanol. Transfer from the gels to the activated membranes was done in Trans-slot SD semi-dry transfer cell (Bio-Rad Laboratories) in transfer buffer (Novex® Tris-Glycine Transfer Buffer (25X); Invitrogen). Membranes were blocked with 5% skim milk in PBS-0.05% Tween 20 for one hour and then incubated with an anti-p24 monoclonal antibody (Santa Cruz Biotechnology, Santa Cruz, CA). Horseradish peroxidase-conjugated anti-mouse immunoglobulin G (Santa Cruz Biotechnology, Santa Cruz, CA) was used as a secondary antibody. Membranes were treated with a chemiluminescent substrate (Bio-Rad Laboratories). The bands were visualized and analysed with a Fujifilm LAS-3000 imager (Fujifilm Life Science USA, Stamford, CT).

### Enzyme-linked Immunospot (ELISPOT) Assay for Interferon (IFN)-γ Release

*Ex vivo* measurement of T cell-specific IFN-γ production was performed by an ELISPOT assay as previously described [75], [76]. Briefly, 96-well plates (Multiscreen Millipore, Bedford, MA) were coated overnight at 4°C with 15 µg/ml of anti-IFN-γ monoclonal antibody 1-D1K (Mabtech, Stockholm, Sweden) in coating buffer (Na_2_CO_3_ 0.1 M, pH 9.6). Plates were washed four times and blocked with RPMI-1640/10% FBS. Thawed PBMCs were added to the coated plates for 16–18 h at a final concentration of 10^5^ cells/100 µl. As described above, various virions were also used: AT-2 inactivated NL4-3; NL4-3/ΔRT, AT-2 inactivated NL4-3/ΔRT and AT-2 inactivated NL4-3 with APV. In all cases we initially evaluated different doses (range from 200 ng/ml to 5 µg/ml HIV-1 p24; data not shown). The elicited response was evaluated against the lowest concentration. Additionally, a negative control (RPMI-1640/10% FBS) and a positive control (PHA 2 µg/ml; SIGMA-ALDRICH) were performed. Cells were lysed with PBS/0.05% Tween-20, and wells were incubated for three hours with 1 µg/ml of biotin-labelled, anti-IFN-γ monoclonal antibody 7-B6-1 (Mabtech). To visualize the spot forming foci (IFN-γ-secreting cells), wells were washed six times and treated for one hour with streptavidin-alkaline phosphatase (Mabtech). After this incubation, wells were washed again and 100 µl/well of chromogenic alkaline-phosphatase conjugated substrate (Bio-Rad Laboratories) was added. Spots were clearly visible in less than 30 minutes avoiding direct light exposure. Spot forming cells (SFC) were counted using an AID ELISPOT reader (Autoimmun Diagnostika GmHb, Strassberg, Germany). Results were expressed as SFC/10^6^ PBMC after subtracting the background. The positivity threshold for each construct or antigen was defined as above 50 SFC/10^6^ PBMC and at least twice that of the control medium.

### Statistical Analysis

Data analysis and comparisons for the different parameters were made using parametric (paired t test) or non-parametric tests (Wilcoxon signed rank test), as appropriate. For all analyses, the level of significance was set at p<0.05. Statistical analysis was performed using the GraphPad Software, (GraphPad Prism version 5.00, San Diego California USA).
